# Innate preference hierarchies coupled with adult experience, rather than larval imprinting or transgenerational acclimation, determine host plant use in *Pieris rapae*

**DOI:** 10.1002/ece3.7018

**Published:** 2020-12-08

**Authors:** Hampus Petrén, Gabriele Gloder, Diana Posledovich, Christer Wiklund, Magne Friberg

**Affiliations:** ^1^ Department of Biology Lund University Lund Sweden; ^2^ CMPG Laboratory for Process Microbial Ecology and Bioinspirational Management (PME&BIM) Department of Microbial and Molecular Systems (M2S) KU Leuven Leuven Belgium; ^3^ Department of Zoology Stockholm University Sweden

**Keywords:** anticipatory epigenetic effects, Hopkins’ host selection principle, host plant specialization, larval performance, maternal effects, oviposition preference

## Abstract

The evolution of host range drives diversification in phytophagous insects, and understanding the female oviposition choices is pivotal for understanding host specialization. One controversial mechanism for female host choice is Hopkins’ host selection principle, where females are predicted to increase their preference for the host species they were feeding upon as larvae. A recent hypothesis posits that such larval imprinting is especially adaptive in combination with anticipatory transgenerational acclimation, so that females both allocate and adapt their offspring to their future host. We study the butterfly *Pieris rapae*, for which previous evidence suggests that females prefer to oviposit on host individuals of similar nitrogen content as the plant they were feeding upon as larvae, and where the offspring show higher performance on the mother's host type. We test the hypothesis that larval experience and anticipatory transgenerational effects influence female host plant acceptance (no‐choice) and preference (choice) of two host plant species (*Barbarea vulgaris* and *Berteroa incana*) of varying nitrogen content. We then test the offspring performance on these hosts. We found no evidence of larval imprinting affecting female decision‐making during oviposition, but that an adult female experience of egg laying in no‐choice trials on the less‐preferred host *Be. incana* slightly increased the *P. rapae* propensity to oviposit on *Be. incana* in subsequent choice trials. We found no transgenerational effects on female host acceptance or preference, but negative transgenerational effects on larval performance, because the offspring of *P. rapae* females that had developed on *Be. incana* as larvae grew slower on both hosts, and especially on *Be. incana*. Our results suggest that among host species, preferences are guided by hard‐wired preference hierarchies linked to species‐specific host traits and less affected by larval experience or transgenerational effects, which may be more important for females evaluating different host individuals of the same species.

## Graphical abstract

The importance of nongenetic inheritance through transgenerational acclimation has received increasing attention. Very few studies have investigated the role of transgenerational effects in the adult host plant preference and larval performance of phytophagous insects, even though these traits are explaining host‐driven diversification in this hyperdiverse group.

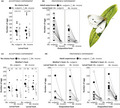

## 1 INTRODUCTION

The evolution of diet breadth is pivotal for the organization of diversity, both through niche partitioning (Schoener 1974) and through ecologically driven diversification (Nosil, 2012). Most phytophagous insects, one of the most common life forms on earth, are host specialists, feeding on a limited number of plant species (Schoonhoven et al., 2005). The host range is determined both by the larval ability to develop on a plant, and by the female propensity to oviposit on that plant. Often, the larval diet breadth is wider than the female host plant preference (Friberg et al., 2015; Wiklund 1975), which means that understanding the female oviposition choice is central for understanding the evolution of host specialization. Host plant preference evolution can cause shifts or expansions in the host use of local populations, which may lead to divergent specialization onto different host plant species and subsequent speciation (e.g., Futuyma & Moreno, 1988; Janz & Nylin, 2008; Janz et al., 2006; Matsubayashi et al., 2010).

Traditionally, the overwhelming majority of specialized phytophagous insects has been explained by the preference‐performance hypothesis, which posits that females that prefer to oviposit on plants that maximize the performance of their offspring are favored by selection (Gripenberg et al., 2010; Thompson & Pellmyr, 1991). Often, however, the female preference hierarchy among potential hosts deviates substantially from the larval growth performance on these plants (Gripenberg et al., 2010; Thompson, 1988), indicating that alternative selection pressures (*cf*. Vidal & Murphy, 2018) and phenotypic plasticity affect the female host plant choice. Indeed, a female's physiological status, such as her size and fecundity (Jaenike, 1978; Schäpers et al., 2017), her current egg load (Berger et al., 2012; Courtney et al., 1989), and the size of a nutrient‐rich spermatophore provided by the male (Schäpers et al., 2017), all may affect female selectivity in the interaction with putative host plant species and individuals. Females also can show experience‐based host preferences by sampling their environment and directing their oviposition toward a locally abundant host (Papaj & Prokopky, 1989; Rausher, 1978). Hence, host plant preference variation among conspecific females or populations could be explained by hard‐wired local genetic variation (Singer & McBride, 2012), by the female's internal status (Berger et al., 2012; Jaenike, 1978; Schäpers et al., 2017), or as a plastic response to local variation in the host community (Fox & Morrow, 1981; Papaj & Prokopky, 1989; Wiklund et al., 2017).

A more controversial aspect of learning is the idea that a female's experience from the larval stage should influence her oviposition preference as an adult (Hopkins, 1917). This “Hopkins' Host Selection Principle” (HHSP) states that an ovipositing female should show a preference for the same type of plant that she consumed as a larva (Barron, 2001), since her successful development into adulthood is evidence for the suitability of that particular host under the local ecological circumstances. For long, empirical evidence supporting HHSP was lacking (Jaenike, 1983; Janz et al., 2009; Kerpel & Moreira, 2005; Rojas & Wyatt, 1999; Tabashnik et al., 1981; Wiklund, 1975; Wiklund et al., 2017), which made Barron (2001) proclaim the death of HHSP. However, during recent decades, an increasing number of studies have found evidence for an impact of larval experience on adult female host preference, which has led to a resurged interest in a potential general importance for HHSP (Akhtar & Isman, 2003; Akhtar et al., 2009; Anderson et al., 1995; Anderson, Sadek, Larsson, Hansson, & Thöming, 2013; Cahenzli et al., 2015; Chow et al., 2005; Facknath & Wright, 2007; Kemp, 2019; Moreau et al., 2008; Olsson et al., 2006; Rietdorf & Steidle, 2002; Thöming et al., 2013). Yet, several aspects of HHSP remain elusive, including the mechanism behind the retention of memories through the metamorphosis of holometabolous insects, the biological meaning of the patterns detected, and the adaptive value of a HHSP‐driven female host plant preference.

Support for HHSP has been obtained using artificial diets (Olsson et al., 2006), aversive chemicals (Chow et al., 2005), different plant species (Thöming et al., 2013), different cultivars (Moreau et al., 2008), and conspecific plants differing in nitrogen content (Cahenzli et al., 2015). The biological relevance of, and differences between, these different types of larval experiences requires further research emphasis. It has been suggested that HHSP would be adaptive when the temporal heterogeneity of host plant availability is low or predictable, and when spatial heterogeneity is high and gene flow between patches with different host plants moderate (Janz et al., 2009; Thöming et al., 2013). Further, the use of larval experience, instead of adult learning, should be beneficial when the cost of adult learning is comparatively high (Janz et al., 2009), which has been argued to often be the case for generalized species (Anderson et al., 2013; Bernays, 2001; Petit et al., 2015). Cahenzli et al. (2015) suggested that HHSP is generally only adaptive in combination with anticipatory transgenerational acclimation (Uller et al., 2013), so that females both allocate and adapt their offspring to the same type of host they experienced as larvae. In their study, the butterfly *Pieris rapae* (Lepidoptera: Pieridae) was reared on *Brassica oleracea* convar. *capitata* (Brassicaceae) from either high‐ or low‐nitrogen treatments. Females reared on high‐nitrogen host plants preferred to also oviposit on high‐nitrogen hosts, whereas females reared on low‐nitrogen host plants increased their relative preference for low‐nitrogen hosts (Cahenzli et al., 2015). The evidence also suggested transgenerational acclimation, with offspring of females reared on low‐nitrogen *Br. oleracea* growing larger than the offspring of females reared on high‐nitrogen *Br. oleracea* when reared on low‐nitrogen host plants. Additionally, these second‐generation females preferred to oviposit on low‐nitrogen host plants as adults. Cahenzli et al. (2015) therefore suggested that such transgenerational effects coupled to female host plant preferences establishing through HHSP could be important drivers of host plant shifts among plant species of different nutrient composition, and thus affect the early stages of the evolution of host specialization. In this study, we test whether the *P. rapae* host utilization is influenced by larval experience and anticipatory transgenerational acclimation also when females are choosing between host plant species that show natural differences in nitrogen content, as well as in other physiological traits, in a series of larval rearing and adult host plant preference experiments.

First, we establish that two natural host plants of *P. rapae*, *Barbarea vulgaris* and *Berteroa incana*, differ in nitrogen content. Thereafter, we attempt to disentangle effects of adult learning, larval experience, and transgenerational acclimation on the host plant preference of *P. rapae* when choosing between host plant species of different nitrogen content. Finally, we test to what extent the larval host of the previous generation affects larval performance on two host plant species varying in nitrogen content.

## 2 METHODS

*Pieris rapae* is a widespread butterfly species occurring across large parts of the world that flies in two to three generations per year in temperate environments (Eliasson et al., 2005). Most eggs are laid on plants from the Brassicaceae family, but *P. rapae* also uses several non‐Brassicaceae species (e.g., *Cleome hassleriana*, *Reseda odorata*, *Tropaeolum majus*) as hosts (Friberg et al., 2015). In this study, we used two Brassicaceae species as hosts, *Ba. vulgaris* and *Be. incana*. *Pieris rapae* females have been shown to prefer ovipositing on *Ba. vulgaris* over *Be. incana*, and larvae grow faster and larger on the preferred species (Friberg et al., 2015).

### 2.1 Plant growth conditions and nitrogen content

Seeds from *Ba. vulgaris* were collected from multiple seed families (50+) from a population in Stockholm, Sweden (59.3663°N, 18.0764°E), and from *Be. incana* from 15 seed families from a population in Uppsala, Sweden (59.8385°N, 17.6323°E). Plants grown from these seeds were used for all experiments. In the experiment on the second generation of butterflies (see below), the larval food plants were supplemented with 30 *Be. incana* individuals that were collected in the field in Stockholm (59.3606°N, 18.0556°E), repotted and kept under the same greenhouse conditions as the greenhouse grown plants. These plants were chosen to be of the same phenology status as the greenhouse grown *Be. incana*.

Plants were kept at an 18 hr day length at approximately 20°C during day (light intensity of ~ 300 μEm^‐2^s^‐1^), and 15°C during nighttime under standard greenhouse conditions. Plants were grown in 6 × 6 × 7 cm plastic pots with a soil mixture consisting of one third 2–6 mm LECA (Saint‐Gobain Byggprodukter AB Sweden), two thirds potting soil (“Yrkes‐Plantjord” Weibulls Horto AB, Sweden), with a thin top layer of sowing soil (“Plugg och Såjord,” Weibulls Horto AB, Sweden) in multiple cohorts, and were presented to butterflies in the pots at approximately the same phenological stage (3–4 weeks old). Previous studies have shown that plant age and whether the plant is still attached or a cutting can affect the oviposition propensity in *Pieris* females (Friberg & Wiklund, 2016). Plants were watered three times a week, twice with only water and once with very moderate levels of fertilizer (N:P:K = 3:1:5, 2ml/l water). We measured the nitrogen content of 102 *Be. incana* and 93 *Ba. vulgaris* individuals, divided among four sowing‐date cohorts at the phenology stage when these plants were presented to the butterflies. We dried leaf samples at 60°C. Plant nitrogen content was analyzed using a Carbon‐Hydrogen‐Nitrogen (CHN) analyzer (Costech ECS (Elemental Combustion System) 4,010).

### 2.2 Butterfly preference and performance experiments

The laboratory population of *P. rapae* descended from a parental generation of 79 eggs collected from *Ba. vulgaris* plants around the village of Vejbystrand, southern Sweden (56.3116°N, 12.7693°E) in autumn 2018. In the laboratory, the newly hatched larvae were transferred to the host plant *Alliaria petiolata* (Brassicaceae) and reared until adult eclosion. We used *A. petiolata* as host in this first laboratory generation to mitigate that any transgenerational effects already acting upon the wild‐caught individuals would affect the results. Adults were released into a communal mating cage, including *A. petiolata* for egg laying. Newly hatched larvae were haphazardly chosen to be reared singly on *Ba. vulgaris* or *Be. incana* until pupation in a 22:2 hr light:dark cycle (light source 18 W Sylvana Gro‐Lux lamps) and a constant temperature of 23°C. Newly eclosed adults were kept at 10°C for up to three days until a sufficient amount of individuals had eclosed to allow for mating. The butterflies were placed in a 0.8 × 0.5 × 0.5 m cage, and females were allowed to mate once with a virgin male. Matings occurred both between individuals that had fed on the same and different host species as larvae. The day after mating, females were released into individual cages measuring 0.8 × 0.5 × 0.5 m for oviposition trials. Cages were placed at room temperature (~20°C) and lit by natural daylight from windows with additional lighting from a lamp above each cage (Philips HQIL 400‐W mercury halogen). Each cage included either a potted *Ba. vulgaris* or a potted *Be. incana*, presented to the female close to the cage ceiling (Friberg & Wiklund, 2019). Eggs were counted daily, and the no‐choice acceptance trials lasted for three consecutive days. Thus, females reared on the different host plants either had access to only *Ba. vulgaris* or only *Be. incana* during the first three egg‐laying days.

On the fourth day, we initiated preference trials (*cf*. Schäpers et al., 2017) by providing females a choice between ovipositing on either *Ba. vulgaris* or *Be. incana*. We varied the position of the different plant species in the cage among females and among days. The preference experiment lasted for three days, and eggs were counted daily. Hence, a female reared on either *Ba. vulgaris* or *Be. incana* either met a *Ba. vulgaris* or a *Be. incana* host for three days and then spent three days with access to both host plants. Thereby, we could test for effects of both larval host plant and adult imprinting on the preference for either plant species. Previous work shows that *Pieris rapae* females lay between 150 and 600 eggs on a preferred host plant over the course of a week after mating (Friberg et al., 2015).

To test for transgenerational effects on female host preference and larval performance (Cahenzli et al., 2015), we reared a subset of the offspring (*n* = 287 larvae) of 16 females that were reared on either *Ba. vulgaris* or *Be. incana* in the same rearing conditions as above (22:2 hr light:dark cycle, 23°C). We measured larval development time (days from newly hatched larva to pupa), adult weight upon eclosion (mg) and growth rate (the 10‐logarithm of adult weight/development time). Eclosing adults were mated, and mated females were undergoing the same treatment as their mothers, that is, first meeting either *Ba. vulgaris* or *Be. incana* for three days, and then facing a choice between the two plant species for the next three days of egg laying.

### 2.3 Statistical analysis

All statistical tests were performed in R 3.6.0 (R Core Team, 2019). Linear models were constructed as necessary to meet assumptions of normality and homoscedasticity in each case (see below).

First, we performed an ANOVA to test whether the nitrogen content differed between the two host plant species (*Ba. vulgaris* or *Be. incana*) and the four plant cohorts. We also included the interaction term between host plant and cohort. Nitrogen content was log‐transformed prior to the analysis.

Second, for each generation, we examined whether the acceptance of the host plant by the adult butterfly in conditions of no‐choice was influenced by the host plant species that the female fed on at the larval stage. We used the total number of eggs laid by each female (across the three days of no‐choice trials) as the response variable. We included as main effects the larval host plant species of the male the female was mating with, the larval host plant species of the female, the no‐choice oviposition host and the interaction between the female larval host plant and the oviposition host. We then stepwise simplified the models by removing interactions and main effects and used Akaike's Information Criterion (AIC) to evaluate the resulting model outputs. We included the larval host of the male as a factor, because previous studies have shown that *P. rapae* grows larger on *Ba. vulgaris* than on *Be. incana* (Friberg et al., 2015), that larger *Pieris* males produce larger spermatophores (Wiklund & Kaitala, 1995), and that the size of the male mating partner may affect female egg‐laying propensity (Schäpers et al., 2017).

In the second generation, we additionally included in the model the host plant species the mother was feeding on. Thus, in the most complex models, we included as main effects the larval host plant species of the male the female was mating with, the mother's host plant, the larval host plant species of the female, the no‐choice oviposition host and either the interaction between the female larval host plant and the oviposition host or the interaction between the mother's host and the oviposition host. We then ran a series of stepwise reduced models and used Akaike's Information Criterion (AIC) to evaluate the model outputs. Thereby, we could identify potential effects of larval host experience and the mother's host experience on female host plant acceptance. Due to unequal variances among groups, we used the White‐adjusted ANOVA (II) in the R package car (Fox & Weisberg, 2019).

For both generations, the preference of females between the two host plants could not be tested using the number of eggs laid on each host because only few females laid eggs on *Be. incana* in the preference trials. We therefore transformed this variable into a binary response variable (eggs on *Be. incana* or not) and tested for the effect of the larval food plant of the female and her male mating partner, the plant to which each female was exposed to in the no‐choice trial and the interaction between the female larval host plant and the no‐choice host. The presence or absence of eggs on *Be. incana* was modeled using a generalized linear model with a binomial error distribution and logit as link function. In the second generation, we again additionally included in the model the host plant species the mother was feeding on to identify transgenerational effects on female host preference. Again, we used model reduction and AIC analyses to evaluate and select the models best fitting the respective datasets.

Finally, we asked whether larval performance was affected by the larval host plant experienced by their mothers. We used the development time (days from egg to adult), adult weight (mg), and growth rate (logarithm of adult weight/development time) of the offspring of females from the first generation as response variables in separate linear mixed models, using the R package nlme (Pinheiro et al., 2017), controlling for heteroscedasticity among groups using the weights function. Each model initially had sex, the mother's host plant, larval host plant and all interactions as factors and the larval family as random factor. The sex of the larvae did not affect either of the response variables either as a stand‐alone factor or in any interaction and was therefore removed from the final models.

## 3 RESULTS

The mean nitrogen content of *Be. incana* plants (2.80 ± 0.15 [mean ± SE] g nitrogen/100 g dry mass) was consistently higher than the mean nitrogen content of *Ba. vulgaris* plants (1.65 ± 0.12 g nitrogen/100 g dry mass) (F_1,187_ = 147.6, *p* < .001), but varied with plant cohort (F_3,187_ = 99.2, *p < *.001). One of the four cohorts showed significantly higher nitrogen content in both host species, but the difference between *Ba. vulgaris* and *Be. incana* was similar to the other cohorts (Supporting Information: Figure S1) (linear model: Plant Species x Plant Cohort F_3,187_ = 1.77, *p* = .15).

**Figure 1:**
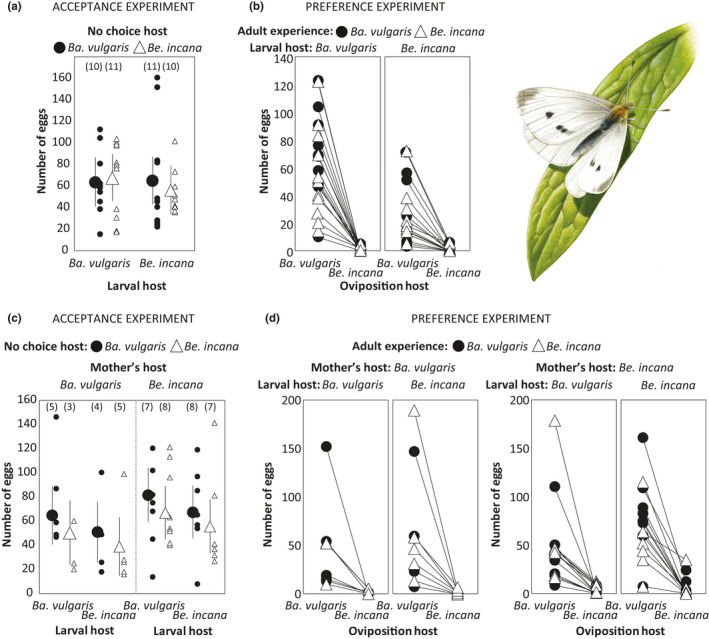
The results of the first (a,b) and second (c,d) generations of host plant acceptance (a,c) and host plant preference experiments (b,d) on *Pieris rapae* females (top right). Host acceptance was independent of both larval host and oviposition host in both generations (a,c), whereas host plant preference was strongly biased towards *Barbarea vulgaris* in both generations (b,d) and not affected by the larval host. We found no effects of the mother's host plant on either host plant acceptance (c), or host plant preference (d). In (a) and (c), large symbols represent means and 95% confidence intervals. Numbers in parentheses in (a) and (c) denote sample sizes. Illustration: Richard Lewington

In both generations, females laid similar numbers of eggs on both host plant species in the no‐choice acceptance trials (Figure 1a,c), but strongly preferred *Ba. vulgaris* over *Be. incana* in the choice preference trials (Figure 1b,d). The female larval host plant experience, or its interaction with the no‐choice host, had no significant impact on her acceptance of a host (the no‐choice trials) (Table S1–S2, Figure 1a,c) in any of the two female generations, and the mother's host plant did not affect the female acceptance of the host plants in the second generation (Table S2). In the first generation, the model best fitting the data according to AIC was the null model (AIC = 419.81) followed by the model only including no‐choice host (AIC = 421.77), which did not have a statistically significant effect on female acceptance (F_1,40_ = 0.034, *p* = .85) (see Supporting information Table S1–S2 for full model analyses). Similarly, in the second generation, we found no significant effects of any factor, but the best model according to AIC included all main effects (AIC = 417.83), that is, male host plant (F_1,36_ = 0.26, *p* = .61), mother's host plant (F_1,36_ = 2.29, *p* = .14), the female host plant (F_1,36_ = 0.15, *p* = .70), and the no‐choice host (F_1,36_ = 0.41, *p* = .52). This model presented a substantially better fit than the null model (AIC = 472.60).

In the choice experiment, the females strongly preferred to oviposit on *Ba. vulgaris* (Figure 1). In fact, only 12 of 42 (29%) females in the first experimental generation and 25 of 47 (53%) females in the second experimental generation oviposited on *Be. incana* in the preference choice trials (Table 1), whereas all females oviposited on *Ba. vulgaris*. The host plant preference, measured as the propensity to lay any eggs on *Be. incana,* was not significantly affected by either the host plant of the male or the female in any of the generations and not by the mother's host plant in the second experiment (Table S3–S4). In the first generation, the null model (without any factors) showed the lowest AIC value (AIC = 52.25) followed by the model including only no‐choice host (AIC = 52.36; χ^2^_1_ = 1.89, *p* = .17). In the second generation, females that had been ovipositing on *Be. incana* in the preceding no‐choice experiment showed a significantly higher propensity to oviposit on *Be. incana* also in the choice experiment (χ^2^_1_ = 8.01, *p* = .0047; Table 1), and the model including only this factor showed the lowest AIC score (AIC = 60.96). This model presented a substantially better fit than the null model (AIC = 66.96).

**Table 1:** The number of *Pieris rapae* females that oviposited on *Berteroa incana* in the choice experiment

Generation	No‐choice host	Eggs on *Berteroa incana*	
		Yes	No
1	*Barbarea vulgaris*	4	17
	*Berteroa incana*	8	13
2	*Barbarea vulgaris*	8	16
	*Berteroa incana*	17	6

**Figure 2:**
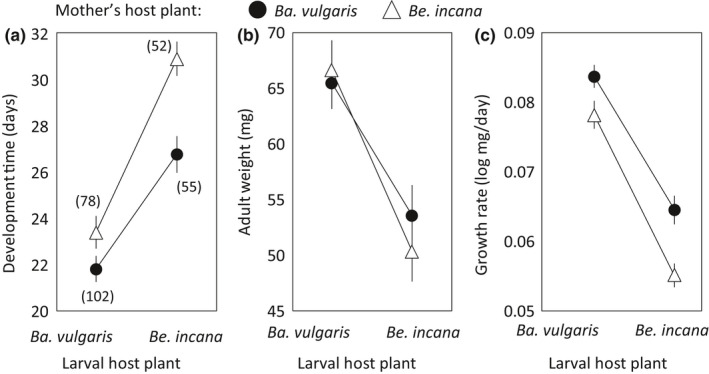
The results from the *Pieris rapae* larval performance trials showing the impact of larval host plant and the host plant of a female's mother (*Barbarea vulgaris* and *Berteroa incana*) on the larval development time (a), adult weight (b), and the resulting growth rate (c). Shown are means (adjusted for family effects) and 95% confidence intervals. Numbers in parentheses in (a) denote sample sizes

Larvae grew faster (χ^2^_1_=728.2, *p* < .001), larger (χ^2^_1_=137.9, *p* < .001), and had a shorter development time (χ^2^_1_=551.4, *p* < .001) on *Ba. vulgaris* than on *Be. incana* (Table 2, Figure 2). For development time (χ^2^_1_=37.8, *p* < .001) and growth rate (χ^2^_1_=49.3, *p* < .001), there was a significant transgenerational effect, because larvae with mothers that had developed on *Be. incana* grew slower and had a longer development time than larvae descending from mothers that had developed on *Ba. vulgaris* (Table 2, Figure 2). These negative transgenerational effects on development time and growth rate were strongest when larvae developed on *Be. incana* (Figure 2), as indicated by a significant interaction effect between the mother's host plant and the larval host (Development time: χ^2^_1_ = 20.2, *p* < .001; Growth rate: χ^2^_1_ = 5.85, *p* = .016).

**Table 2:** Results (Analysis of Deviance) from linear mixed models testing the effect of the mother's host, larval host and their interaction on the development time, adult weight and growth rate of *Pieris rapae* larvae reared on either *Barbarea vulgaris* or *Berteroa incana*. Significant effects are indicated in bold

	Development time			Adult weight			Growth rate		
	χ^2^	*df*	*P*	χ^2^	*df*	*P*	χ^2^	*df*	*P*
Mother's host (MH)	37.8	1	**<0.001**	0.36	1	0.55	49.3	1	**<0.001**
Larval host (LH)	551.4	1	**<0.001**	137.9	1	**<0.001**	728.2	1	**<0.001**
MH * LH	20.2	1	**<0.001**	3.22	1	0.073	5.85	1	**0.016**

## 4 DISCUSSION

The first major conclusion drawn from our results is that adult experience has a small effect on female host preference in *P. rapae* butterflies (Table 1), whereas there is no support for either larval experience (HHSP) or anticipatory transgenerational acclimation to affect the female propensity to oviposit on a certain host plant species (Figure 1). The second major conclusion is that the only transgenerational effect detected was negative and regarded larval performance. The offspring of mothers that had been feeding on *Be. incana* grew to similar size as offspring of mothers that had been feeding on *Ba. vulgaris*, but the offspring from mothers reared on *Be. incana* grew slower and thus had lower growth rates on both host plants, and disproportionally lower growth rates on *Be. incana* (Figure 2, Table 2). Hence, our results do not lend support to the hypothesis that larval experience (HHSP) coupled to transgenerational acclimation to host plant nitrogen levels impact female choice and preadapt larvae to different host plant species.

The lack of evidence for larval experience affecting female host plant acceptance or preference contrasts to recent studies finding positive effects of larval host conditions on adult female oviposition patterns (HHSP) (Akhtar & Isman, 2003; Akhtar et al., 2009; Anderson et al., 1995, 2013; Cahenzli et al., 2015; Chow et al., 2005; Facknath & Wright, 2007; Kemp, 2019; Moreau et al., 2008; Olsson et al., 2006; Rietdorf & Steidle, 2002; Thöming et al., 2013). Furthermore, even though adult learning had some impact on female host preference, as has previously been shown for other aspects of female host use in *P. rapae* (Snell‐Rood & Papaj, 2009; Traynier, 1986), the vast majority of eggs were laid on *Ba. vulgaris* when females were given a choice, indicating that their innate host plant rank order is not easily altered even by previous adult experience. Contrary to a previous study on *P. rapae* (Cahenzli et al., 2015), we found no evidence of transgenerational acclimation in female host plant preference, because females of the second generation were not more inclined to oviposit on the host that their mothers had experienced as larvae.

An increasing number of studies on diverse organisms in different ecological circumstances are reporting examples of anticipatory transgenerational acclimation (Yin et al., 2019), but its role for ecological adaptation is debated (Uller et al., 2013), and its adaptive potential is contingent on the environmental predictability across generations (Colicchio & Herman, 2020). Lepidopterans in general, and butterflies in particular, have been pointed out as especially suitable targets for studies of transgenerational effects, because of their high sensitivity to environmental variation within generations, their multivoltinism and seasonal polyphenism, and their well‐defined resources in terms of host plants (Woestmann & Saastamoinen, 2016). Yet, only very few studies have investigated a potential role of anticipatory transgenerational effects on larval performance in butterflies (Cahenzli & Erhardt, 2013; Cahenzli et al., 2015; Rotem et al., 2003; Woestmann & Saastamoinen, 2016). In Cahenzli et al. (2015), *P. rapae* larvae grew larger on *Br. oleracea* plants of low‐nitrogen content if their mothers had been experiencing similar conditions as larvae. By contrast, in our study, larvae grew faster on both host plants if their mothers had been feeding on the preferred, and more suitable, host *Ba. vulgaris* than if their mothers had been growing on *Be. incana*. In fact, offspring of mothers reared on *Be. incana* grew disproportionally slower when reared on *Be. incana*, indicating a negative transgenerational effect. Previous studies have shown that female status may affect egg provisioning in other lepidopterans (e.g., Diss et al., 1996; Gibbs et al., 2010), which could be a candidate mechanism also for explaining why the offspring of females reared on *Be. incana* showed slower growth rates than the offspring of females reared on *Ba. vulgaris*. In any case, our data do not support that larvae are preadapted through transgenerational acclimation to perform especially well on the host of their mother, and instead reveal negative transgenerational effects that need to be controlled for in future studies of larval performance.

The contrasting results of this study and previous work (Cahenzli et al., 2015) may have multiple, nonmutually exclusive, explanations. We tried to control for stochastic effects by testing the impact of larval host plant on female host acceptance and preference in both generations, as a recent study has indicated substantial variation in host plant acceptance among different cohorts of *Pieris* butterflies experiencing similar experimental conditions (Schäpers et al., 2017).

The different results between our study and Cahenzli et al. (2015) could theoretically reflect local differences between Swedish and Swiss *P. rapae* populations, driven by local selection for or against the impact of larval experience and transgenerational acclimation influencing host choice. In Sweden, the first generation of the year normally occurs in coastal and agricultural areas and likely constitutes a mix of individuals that have overwintered locally and individuals that have dispersed from central Europe. Subsequent generations disperse inland and northwards, especially during warm summers (Eliasson et al., 2005). The availability of different host plant species is therefore spatially variable, and larval experience will likely provide little information about future host suitability. Potentially, the Swiss population can track their favored habitats and hosts by dispersing into higher altitudes as summer progresses. Under such a scenario, local selection pressures would differ between Swedish and Swiss populations. However, *P. rapae* is a mobile species and may normally move 100–200 km during its lifetime (Eliasson et al., 2005), which would counteract the formation of local adaptations, as suggested by the lack of genetic structure within Europe (Ryan et al., 2019).

More likely, the inconsistent results reflect the different experimental setups between studies. Our experiments focused on variation between different host plant species of different nitrogen content, to test the hypothesis that HHSP coupled with transgenerational effects could facilitate host plant switches, whereas the study by Cahenzli et al. (2015) kept host species constant and focused on comparing the preference and performance on host individuals of varying nitrogen content. When given a choice, the *P. rapae* females strongly preferred *Ba. vulgaris* over *Be. incana*, even though *Be. incana* consistently contained higher levels of nitrogen. Several previous studies have shown that both female preference and larval performance of *Pieris* butterflies increase with nitrogen content, when comparing fertilized/unfertilized plants of the same species (Chen et al., 2004; Hwang et al., 2008; Myers, 1985). Thus, the reduced female preference and larval performance (Friberg et al., 2015) for the nitrogen‐rich *Be. incana* can likely be attributed to species‐specific differences in plant defenses against herbivory. *Pieris* butterflies have evolved mechanisms to metabolize glucosinolates (Agerbirk et al., 2010; Wittstock et al., 2004), which are plant secondary metabolites acting as chemical defense in many Brassicaceae species, and even use glucosinolates as stimuli for oviposition or feeding (Chew & Renwick, 1995; Hopkins et al., 2009). Yet, several studies suggest negative correlations between glucosinolate concentration and larval growth rate in *Pieris* butterflies (Agrawal & Kurashige, 2003; Kos et al., 2012; Okamura et al., 2019). Thus, it is possible that the specific concentration and/or composition of glucosinolates in *Be. incana*, which is different to that of *Ba. vulgaris* (Cole, 1976), potentially combined with the presence of other secondary metabolites (Huang et al., 1993; Renwick & Radke, 1988), reduce the larval growth rate and/or female preference for this plant species. Furthermore, whereas *Ba. vulgaris* leaves are glabrous, *Be. incana* leaves are densely covered in trichomes, potentially generating a physical defense against herbivory, and affecting larval growth. It is thus possible that effects of nitrogen content of the larval host can affect female host preference when evaluating different host individuals that are similar in other traits of importance (Cahenzli et al., 2015), while other aspects of host plant quality are more important for female host choice of plants of highly variable suitability, like *Be. incana* and *Ba. vulgaris*.

Previous work on *Spodoptera littoralis*, which is the species providing most convincing examples of larval‐experience driven female host choice, indicates that females must have been feeding on plants above a certain suitability threshold for HHSP to guide their adult host preference (Lhomme et al., 2018; Thöming et al., 2013). The results from *S. littoralis* support the idea that HHSP is a beneficial strategy only when host plants of sufficient suitability are spatially distributed in a way that the local abundance of such hosts can be predicted from one generation to the next based on the larval food availability (Janz et al., 2009; Thöming et al., 2013). For anticipatory transgenerational acclimation to evolve, however, the major underlying selection pressure is a difference in host plant suitability. Thus, female genotypes that can prepare their offspring for future conditions and thereby mitigate negative effects on offspring performance should be favored by selection. Hence, there may be a disconnect between the circumstances that favor larval‐experience‐based female host choice (HHSP) and anticipatory transgenerational acclimation, which could explain the scarcity of examples of diet driven transgenerational effects in Lepidoptera (Woestmann & Saastamoinen, 2016). In the light of our work and previous studies (Cahenzli et al., 2015; Wiklund et al., 2017), the combination of larval‐experience driven host choice and anticipatory transgenerational acclimation is more likely to be important for within plant species host plant choice, whereas among host species preferences are guided by more hard‐wired preference hierarchies linked to species‐specific host traits. Our study further shows the presence of quite substantial negative transgenerational effects of the mother's host species on larval performance, which need to be taken into account in future studies comparing the fitness of phytophagous species on different putative hosts.

## CONFLICT OF INTEREST

The authors declare that they have no conflict of interest.

## AUTHOR CONTRIBUTION

**Hampus Petrén:** Conceptualization (equal); Data curation (equal); Formal analysis (equal); Investigation (equal); Methodology (equal); Validation (equal); Visualization (equal); Writing‐original draft (equal); Writing‐review & editing (equal). **Gabriele Gloder:** Conceptualization (supporting); Investigation (supporting); Methodology (supporting); Writing‐review & editing (supporting). **Diana Posledovich:** Conceptualization (supporting); Investigation (supporting); Methodology (supporting); Writing‐review & editing (supporting). **Christer Wiklund:** Conceptualization (equal); Investigation (equal); Methodology (equal); Resources (equal); Writing‐review & editing (supporting). **Magne Friberg:** Conceptualization (equal); Data curation (equal); Formal analysis (equal); Funding acquisition (lead); Investigation (supporting); Methodology (equal); Project administration (lead); Resources (lead); Supervision (lead); Visualization (lead); Writing‐original draft (equal); Writing‐review & editing (equal).

## Supporting information

Appendix S1

## Data Availability

Data are available from the Dryad Digital Repository at https://doi.org/10.5061/dryad.9w0vt4bd8.
