# Supplementary material for: Innate preference hierarchies coupled with adult experience, rather than larval imprinting or transgenerational acclimation, determine host plant use in Pieris rapae
Source: Ecol Evol. 2020 Dec 8;11(1):242–51. doi: 10.1002/ece3.7018 (PMC7790653; doi:10.1002/ece3.7018)
Supplement: Supplementary file 1 — Appendix S1 [file ECE3-11-242-s001.docx]

Supporting Information for:

**Innate preference hierarchies coupled with adult experience, rather than larval imprinting or transgenerational acclimation, determine host plant use in *Pieris rapae***

Hampus Petrén, Gabriele Gloder, Diana Posledovich, Christer Wiklund, Magne Friberg

*Supplementary results*


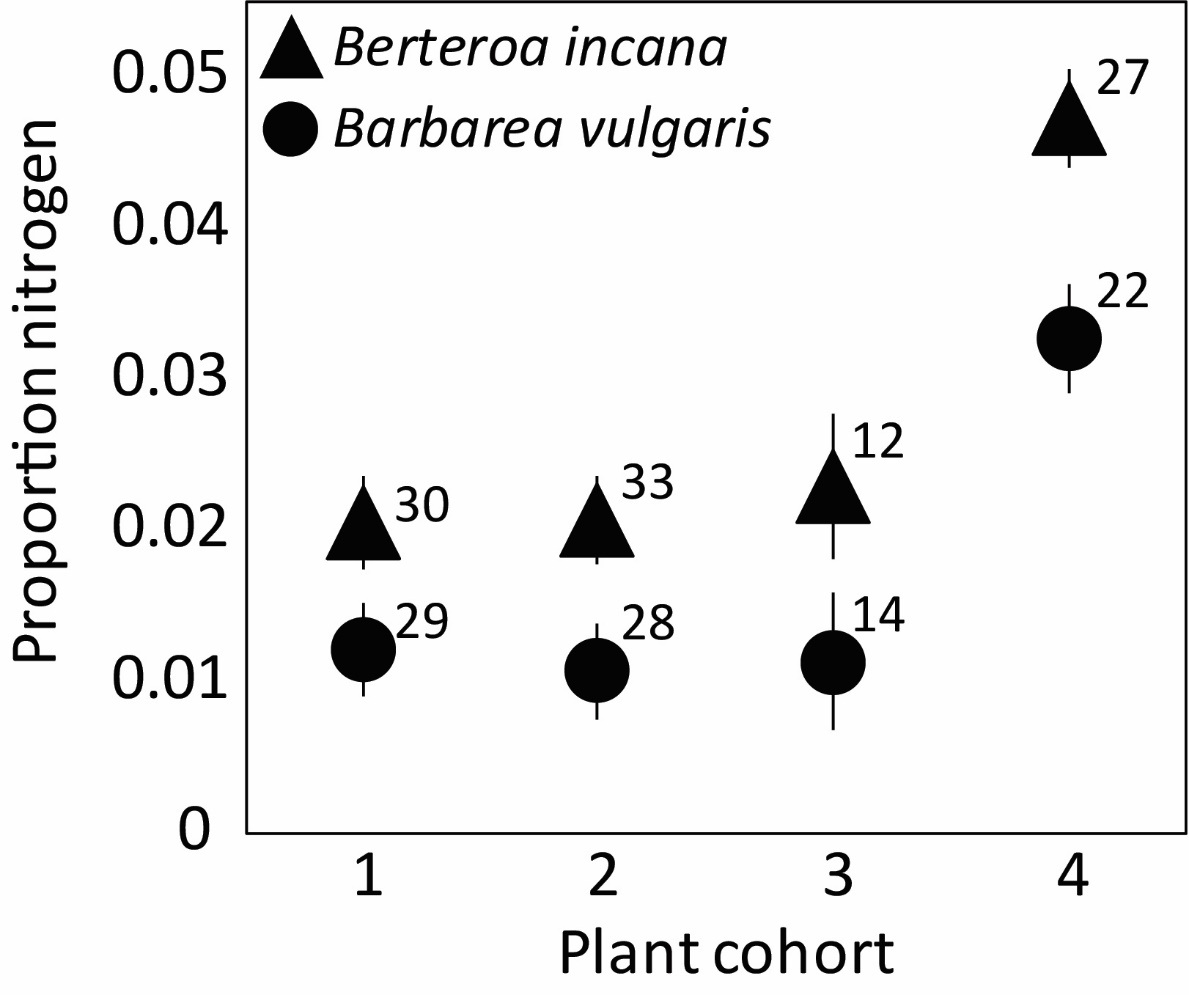


**Figure S1:** The nitrogen content of four different cohorts of *Berteroa incana* (triangles) and *Barbarea vulgaris* (circles), grown under similar conditions. Note the consistent variation between the two species, and the higher nitrogen content of the fourth plant cohort. The reason for the overall higher values of cohort four is unknown, and potential explanations includes mistakes during fertilization and slight differences in the phenological stage of plants used for testing among cohorts. Shown are means ± 95% confidence intervals around the means. Numbers next to symbols denote sample sizes of each group.

We tested the fit of several alternative models for host plant acceptance (no choice experiment) and host plant preference (propensity to oviposit on *Berteroa incana* in the choice experiment) using Akaike’s Information Criterion. We ran separate models for each experiment and generation. In the acceptance experiment, no model produced a lower AIC score than the null-model (no factors) in the first generation (Table S1). In the second generation the model with the lowest AIC score included all main factors (i.e. the host plant of the male mating partner, the mother’s host plant, the female’s larval host plant and the no-choice host plant (Table S2).

In the preference experiment, the model with the lowest AIC score was again the null-model for generation 1 (Table S3). In generation 2, the best model included only the no choice host that the female had experienced during her first three days of egg-laying (Table S4).

**Table S1:** Results from the AIC analysis of models explaining female host plant acceptance in Generation 1. The null model (f) showed the lowest AIC score, followed by a non-significant model including only the no-choice host the female was ovipositing on (e).

| **Generation 1** | a) AIC = 425.88 | | | b) AIC = 424.36 | | | c) AIC = 425.17 | | | d) AIC = 423.56 | | | e) AIC = 421.77 | | | **f) AIC = 419.81** | | |
| --- | --- | --- | --- | --- | --- | --- | --- | --- | --- | --- | --- | --- | --- | --- | --- | --- | --- | --- |
|  | *df* | *F* | *P* | *df* | *F* | *P* | *df* | *F* | *P* | *df* | *F* | *P* | *df* | *F* | *P* |  |  |  |
| Male's host | 1 | 1.07 | 0.31 | 1 | 1.07 | 0.31 |  |  |  |  |  |  |  |  |  |  |  |  |
| Female's host (FH) | 1 | 0.27 | 0.61 | 1 | 0.11 | 0.74 | 1 | 0.45 | 0.50 | 1 | 0.20 | 0.66 |  |  |  |  |  |  |
| No choice host (NH) | 1 | 0.0057 | 0.94 | 1 | 0.040 | 0.84 | 1 | 0.012 | 0.91 | 1 | 0.043 | 0.84 | 1 | 0.034 | 0.85 |  |  |  |
| FH x NH | 1 | 0.39 | 0.54 |  |  |  | 1 | 0.33 | 0.57 |  |  |  |  |  |  |  |  |  |
| Residuals | 37 |  |  | 38 |  |  | 38 |  |  | 39 |  |  | 40 |  |  | 41 |  |  |

**Table S2:** Results from the AIC analysis of models explaining female host plant acceptance in Generation 2. The model with the lowest AIC score included all four, non-significant main factors and no interaction effect (c). The null model (h) showed a substantially higher AIC score.

| **Generation 2** | a) AIC = 419.25 | | | b) AIC = 419.65 | | | **c) AIC = 417.83** | | | d) AIC = 472.68 | | | e) AIC = 473.71 | | | f) AIC = 474.67 | | | g) AIC = 472.98 | | | h) AIC = 472.60 | | |
| --- | --- | --- | --- | --- | --- | --- | --- | --- | --- | --- | --- | --- | --- | --- | --- | --- | --- | --- | --- | --- | --- | --- | --- | --- |
|  | *df* | *F* | *P* | *df* | *F* | *P* | *df* | *F* | *P* | *df* | *F* | *P* | *df* | *F* | *P* | *df* | *F* | *P* | *df* | *F* | *P* | *df* | *F* | *P* |
| Male's host | 1 | 0.14 | 0.71 | 1 | 0.19 | 0.67 | 1 | 0.26 | 0.61 |  |  |  |  |  |  |  |  |  |  |  |  |  |  |  |
| Mother's host (MH) | 1 | 2.47 | 0.12 | 1 | 2.45 | 0.13 | 1 | 2.29 | 0.14 | 1 | 2.21 | 0.14 | 1 | 2.54 | 0.12 | 1 | 2.14 | 0.15 |  |  |  |  |  |  |
| Female's host (FH) | 1 | 0.11 | 0.74 | 1 | 0.10 | 0.75 | 1 | 0.15 | 0.70 | 1 | 1.44 | 0.24 | 1 | 1.16 | 0.29 | 1 | 1.37 | 0.25 |  |  |  |  |  |  |
| No choice host (NH) | 1 | 0.39 | 0.53 | 1 | 0.42 | 0.52 | 1 | 0.41 | 0.52 | 1 | 1.58 | 0.22 | 1 | 1.56 | 0.22 | 1 | 1.52 | 0.22 | 1 | 1.51 | 0.23 |  |  |  |
| MH x NH | 1 | 0.41 | 0.53 |  |  |  |  |  |  |  |  |  | 1 | 0.79 | 0.38 |  |  |  |  |  |  |  |  |  |
| FH x NH |  |  |  | 1 | 0.13 | 0.72 |  |  |  |  |  |  |  |  |  | 1 | 0.01 | 0.91 |  |  |  |  |  |  |
| Residuals | 35 |  |  |  | 35 |  | 36 |  |  | 43 |  |  | 42 |  |  | 42 |  |  | 45 |  |  | 46 |  |  |

**Table S3:** Results from the AIC analysis of models explaining female host plant preference, measured as the propensity to oviposit on *Berteroa incana* in the choice experiment in Generation 1. The null model (g) showed the lowest AIC score, followed by a non-significant model including only the no-choice host the female was ovipositing on in the acceptance experiment (f).

| **Generation 1** | a) AIC = 57.25 | | | b) AIC = 55.32 | | | c) AIC = 56.32 | | | d) AIC = 54.36 | | | e) AIC = 54.25 | | | f) AIC = 52.36 | | | **g) AIC = 52.25** | | |
| --- | --- | --- | --- | --- | --- | --- | --- | --- | --- | --- | --- | --- | --- | --- | --- | --- | --- | --- | --- | --- | --- |
|  | *df* | χ*^2^* | *P* | *df* | χ*^2^* | *P* | *df* | χ*^2^* | *P* | *df* | χ*^2^* | *P* | *df* | χ*^2^* | *P* | *df* | χ*^2^* | *P* | *df* | χ*^2^* | *P* |
| Male's host | 1 | 1.07 | 0.30 | 1 | 1.04 | 0.31 |  |  |  |  |  |  |  |  |  |  |  |  |  |  |  |
| Female's host (FH) | 1 | 0.0005 | 0.98 | 1 | 0.005 | 0.98 | 1 | 0 | 0.95 | 1 | 0 | 0.95 | 1 | 0 | 1 |  |  |  |  |  |  |
| No choice host (NH) | 1 | 1.93 | 0.16 | 1 | 1.93 | 0.16 | 1 | 1.90 | 0.17 | 1 | 1.90 | 0.17 |  |  |  | 1 | 1.89 | 0.17 |  |  |  |
| FH x NH | 1 | 0.07 | 0.80 |  |  |  | 1 | 0.04 | 0.85 |  |  |  |  |  |  |  |  |  |  |  |  |

| **Generation 2** | a) AIC = 63.13 | | | b) AIC = 62.82 | | | c) AIC = 62.31 | | | d) AIC = 61.15 | | | e) AIC = 64.29 | | | f) AIC = 68.56 | | | g) AIC = 68.76 | | | **h) AIC = 60.96** | | | i) AIC = 66.96 | | |
| --- | --- | --- | --- | --- | --- | --- | --- | --- | --- | --- | --- | --- | --- | --- | --- | --- | --- | --- | --- | --- | --- | --- | --- | --- | --- | --- | --- |
|  | *df* | χ*^2^* | *P* | *df* | χ*^2^* | *P* | *df* | χ*^2^* | *P* | *df* | χ*^2^* | *P* |  |  |  | *df* | χ*^2^* | *P* | *df* | χ*^2^* | *P* | *df* | χ*^2^* | *P* | *df* | χ*^2^* | *P* |
| Male's host | 1 | 0.32 | 0.57 | 1 | 0.21 | 0.65 | 1 | 0.20 | 0.65 | 1 | 0.31 | 0.58 |  |  |  |  |  |  |  |  |  |  |  |  |  |  |  |
| Mother's host (MH) | 1 | 0.33 | 0.57 | 1 | 0.33 | 0.57 | 1 | 0.16 | 0.69 | 1 | 0.33 | 0.57 | 1 | 0.36 | 0.55 | 1 | 0.40 | 0.53 |  |  |  |  |  |  |  |  |  |
| Female's host (FH) | 1 | 0.59 | 0.44 | 1 | 0.61 | 0.43 | 1 | 0.59 | 0.44 | 1 | 0.59 | 0.44 | 1 | 0.30 | 0.59 |  |  |  | 1 | 0.20 | 0.65 |  |  |  |  |  |  |
| No choice host (NH) | 1 | 3.88 | **0.049** | 1 | 3.90 | **0.048** | 1 | 3.90 | **0.048** | 1 | 3.90 | **0.048** | 1 | 8.09 | 0.0045 |  |  |  |  |  |  | 1 | 8.01 | **0.0047** |  |  |  |
| MH x FH | 1 | 0.02 | 0.89 |  |  |  |  |  |  |  |  |  |  |  |  |  |  |  |  |  |  |  |  |  |  |  |  |
| MH x NH |  |  |  | 1 | 0.33 | 0.57 |  |  |  |  |  |  |  |  |  |  |  |  |  |  |  |  |  |  |  |  |  |
| FH x NH |  |  |  |  |  |  | 1 | 0.84 | 0.36 |  |  |  |  |  |  |  |  |  |  |  |  |  |  |  |  |  |  |

**Table S4:** Results from the AIC analysis of models explaining female host plant preference, measured as the propensity to oviposit on *Berteroa incana* in the choice experiment in Generation 2. The model including only the no-choice host the female was ovipositing on in the preceding acceptance experiment (h) showed the lowest AIC-score. This factor was the only significant predictor in any of the models. The null model (i) showed a substantially higher AIC score.
